# The genome sequence of the Arran brown, *Erebia ligea* (Linnaeus, 1758)

**DOI:** 10.12688/wellcomeopenres.18115.1

**Published:** 2022-10-13

**Authors:** Konrad Lohse, Alex Hayward, Dominik R. Laetsch, Roger Vila, Kay Lucek, Wellcome Sanger Institute Tree of Life programme, Wellcome Sanger Institute Scientific Operations: DNA Pipelines collective, Tree of Life Core Informatics collective, Darwin Tree of Life Consortium

**Affiliations:** 1 Institute of Evolutionary Biology, University of Edinburgh, Edinburgh, UK; 2 College of Life and Environmental Sciences, Department of Biosciences, University of Exeter, Exeter, UK; 3 Institut de Biologia Evolutiva, CSIC - Universitat Pompeu Fabra, Barcelona, Spain, Spain; 4 Department of Environmental Sciences, University of Basel, Basel, Switzerland

**Keywords:** Erebia ligea, Arran brown, genome sequence, chromosomal, Lepidoptera

## Abstract

We present a genome assembly from an individual male *Erebia ligea* (Arran brown; Arthropoda; Insecta; Lepidoptera; Nymphalidae). The genome sequence is 506 megabases in span. The majority (99.92%) of the assembly is scaffolded into 29 chromosomal pseudomolecules, with the Z sex chromosome assembled. The complete mitochondrial genome was also assembled and is 15.2 kilobases in length.

## Species taxonomy

Eukaryota; Metazoa; Ecdysozoa; Arthropoda; Hexapoda; Insecta; Pterygota; Neoptera; Endopterygota; Lepidoptera; Glossata; Ditrysia; Papilionoidea; Nymphalidae; Satyrinae; Satyrini; Erebiina; *Erebia*; *Erebia ligea* (Linnaeus, 1758) (NCBI:txid111903).

## Background

The Arran brown, *Erebia ligea*, is one of the most widespread species of the genus *Erebia*, occurring from the Russian Kamchatka Peninsula and Japan in eastern Asia ( Dubatolov *et al.*, 1998) to central and northern Europe ( Kudrna *et al.*, 2015). Although the species takes its common name from the Isle of Arran in Scotland, where it was first recorded in 1803, the current and historic presence of this butterfly in the British Isles remains disputed ( Salmon, 1995). The intraspecific phenotypic diversity present throughout the distribution of *E. ligea* has triggered the description of several subspecies ( Dubatolov *et al.*, 1998; Warren, 1937; Zakharova & Tatarinov, 2016), however, a formal biogeographic assessment remains lacking.

*E. ligea* is characterised as a woodland species associated with clearings and meadows, and occurs at relatively low altitudes compared to most other *Erebia* butterflies ( Kleckova *et al.*, 2014). Recorded host plants include a variety of grasses (Poaceae) and sedges ( *Carex*, Cyperaceae). It is univoltine and in some northern localities it is recorded only every second year ( Tolman & Lewington, 2008). Although *E. ligea* is considered a species of Least Concern according to the IUCN Red List (Europe) ( van Swaay *et al.*, 2010), the species can be locally endangered ( Fichefet *et al.*, 2008).

While the first karyotypic analysis suggested that male *Erebia ligea* from Finland have 29 chromosomes ( Federley, 1938), Japanese individuals from Hokkaido were found to have only 28 chromosomes ( Saitoh & Abe, 1997). These values are close to the most common and putatively ancestral chromosomal state for Lepidoptera (n=31; Robinson, 1971), although *Erebia* is one of the most karyologically diverse known genera of butterflies ( Robinson, 1971; de Vos *et al.*, 2020).

## Genome sequence report

**Figure 1.**
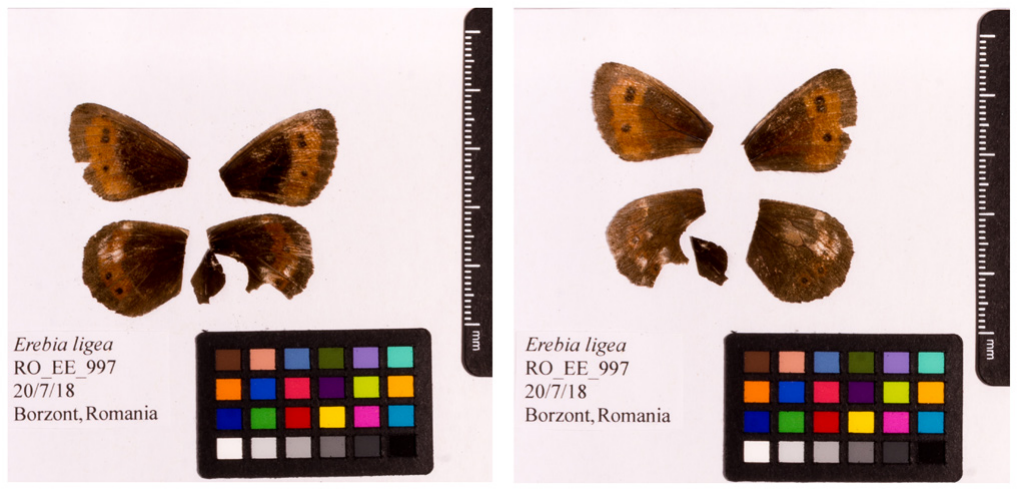
Forewings and hindwings of the male *Erebia ligea* specimen from which the genome was sequenced. Dorsal (left) and ventral (right) surface view of wings from specimen RO_EE_997 (ilEreLige1) from Borzont, Joseni, Harghita, Romania, used to generate Pacific Biosciences, 10X genomics and Hi-C data.

The genome was sequenced from a single male *E. ligea* ( Figure 1) collected from Borzont, Joseni, Harghita, Romania (latitude 46.664, longitude 25.317). A total of 34-fold coverage of Pacific Biosciences single-molecule circular consensus (HiFi) long reads and 63-fold coverage of 10X Genomics read clouds were generated. Primary assembly contigs were scaffolded with chromosome conformation Hi-C data. Manual assembly curation corrected 47 missing/misjoins and removed 10 haplotypic duplications, reducing the assembly length by 3.59% and the scaffold number by 39.39%, and increased the scaffold N50 by 4.29%.

**Figure 2.**
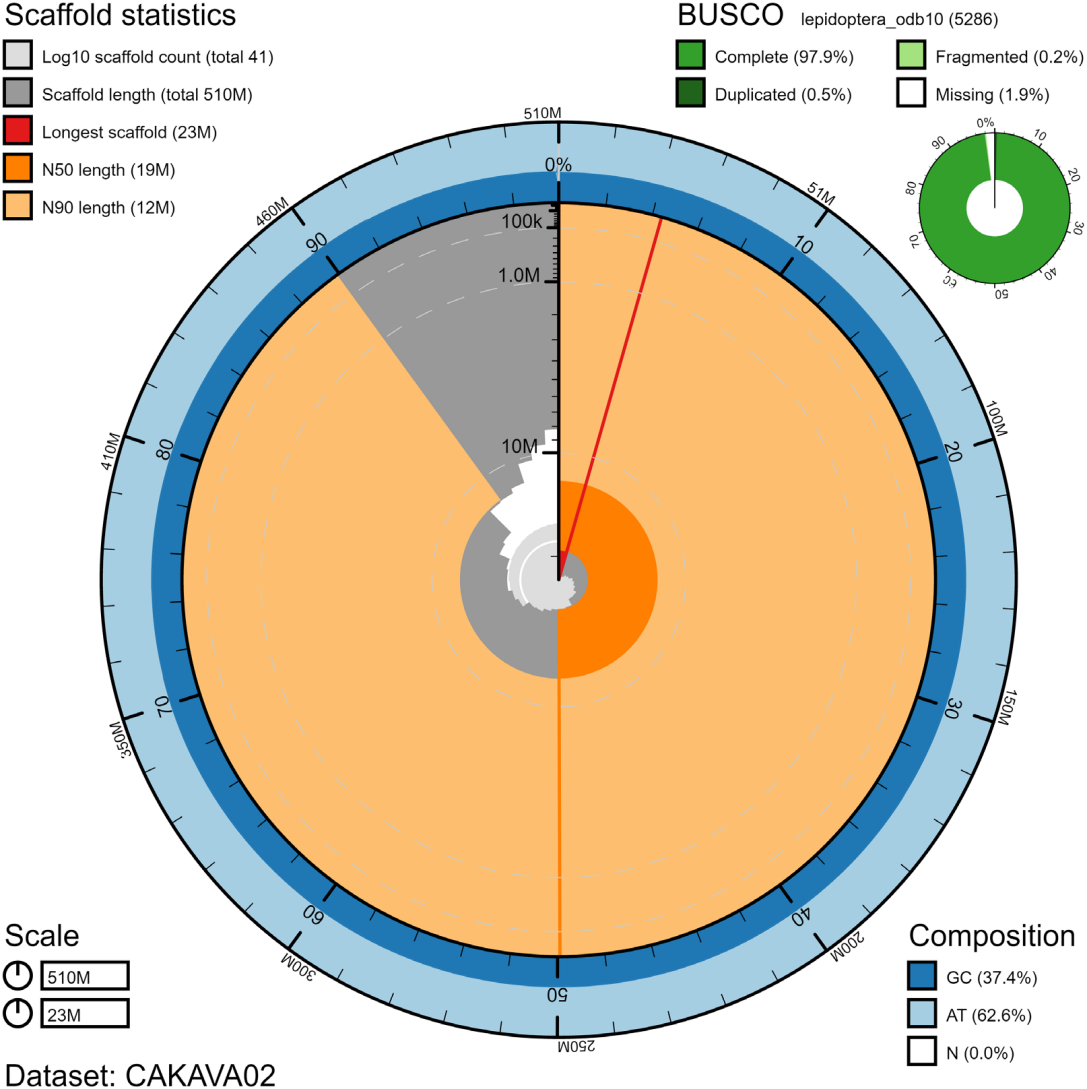
Genome assembly of *Erebia ligea*, ilEreLige1.2: metrics. The BlobToolKit Snailplot shows N50 metrics and BUSCO gene completeness. The main plot is divided into 1,000 size-ordered bins around the circumference with each bin representing 0.1% of the 506,397,422 bp assembly. The distribution of chromosome lengths is shown in dark grey with the plot radius scaled to the longest chromosome present in the assembly (22,722,498 bp, shown in red). Orange and pale-orange arcs show the N50 and N90 chromosome lengths (19,149,538 and 12,368,103 bp), respectively. The pale grey spiral shows the cumulative chromosome count on a log scale with white scale lines showing successive orders of magnitude. The blue and pale-blue area around the outside of the plot shows the distribution of GC, AT and N percentages in the same bins as the inner plot. A summary of complete, fragmented, duplicated and missing BUSCO genes in the lepidoptera_odb10 set is shown in the top right. An interactive version of this figure is available at https://blobtoolkit.genomehubs.org/view/ilEreLige1.2/dataset/CAKAVA02/snail.

The final assembly has a total length of 506 Mb in 40 sequence scaffolds, with a scaffold N50 of 19.1 Mb ( Table 1). The majority, 99.92%, of assembly sequence was assigned to 40 chromosomal-level scaffolds, representing 28 autosomes (numbered by sequence length), and the Z sex chromosome ( Figure 2– Figure 5; Table 2). The assembly has a BUSCO v5.2.2 ( Manni *et al.*, 2021) completeness of 97.9% (single 97.4%, duplicated 0.5%) using the lepidoptera_odb10 reference set (n=5,286). While not fully phased, the assembly deposited is of one haplotype. Contigs corresponding to the second haplotype have also been deposited.

**Table 1.** Genome data for *Erebia ligea*, ilEreLige1.2.

*Project accession data*	
Assembly identifier	ilEreLige1.2
Species	*Erebia ligea*
Specimen	ilEreLige1 (genome assembly, Hi-C)
NCBI taxonomy ID	NCBI:txid111903
BioProject	PRJEB42125
BioSample ID	SAMEA7523313
Isolate information	Male, whole organism
*Raw data accessions*	
PacificBiosciences SEQUEL II	ERR7141799
10X Genomics Illumina	ERR6745725-ERR6745728
Hi-C Illumina	ERR6745729-ERR6745732
*Genome assembly*	
Assembly accession	GCA_917051295.2
Span (Mb)	506
Number of contigs	78
Contig N50 length (Mb)	14.9
Number of scaffolds	40
Scaffold N50 length (Mb)	19.1
Longest scaffold (Mb)	22.7
BUSCO * genome score	C:97.9%[S:97.4%,D:0.5%],F:0.2%, M:1.9%,n:5,286

*BUSCO scores based on the lepidoptera_odb10 BUSCO set using v5.2.2. C= complete [S= single copy, D=duplicated], F=fragmented, M=missing, n=number of orthologues in comparison. A full set of BUSCO scores is available at https://blobtoolkit.genomehubs.org/view/ilEreLige1.2/dataset/CAKAVA02/busco.

**Figure 3.**
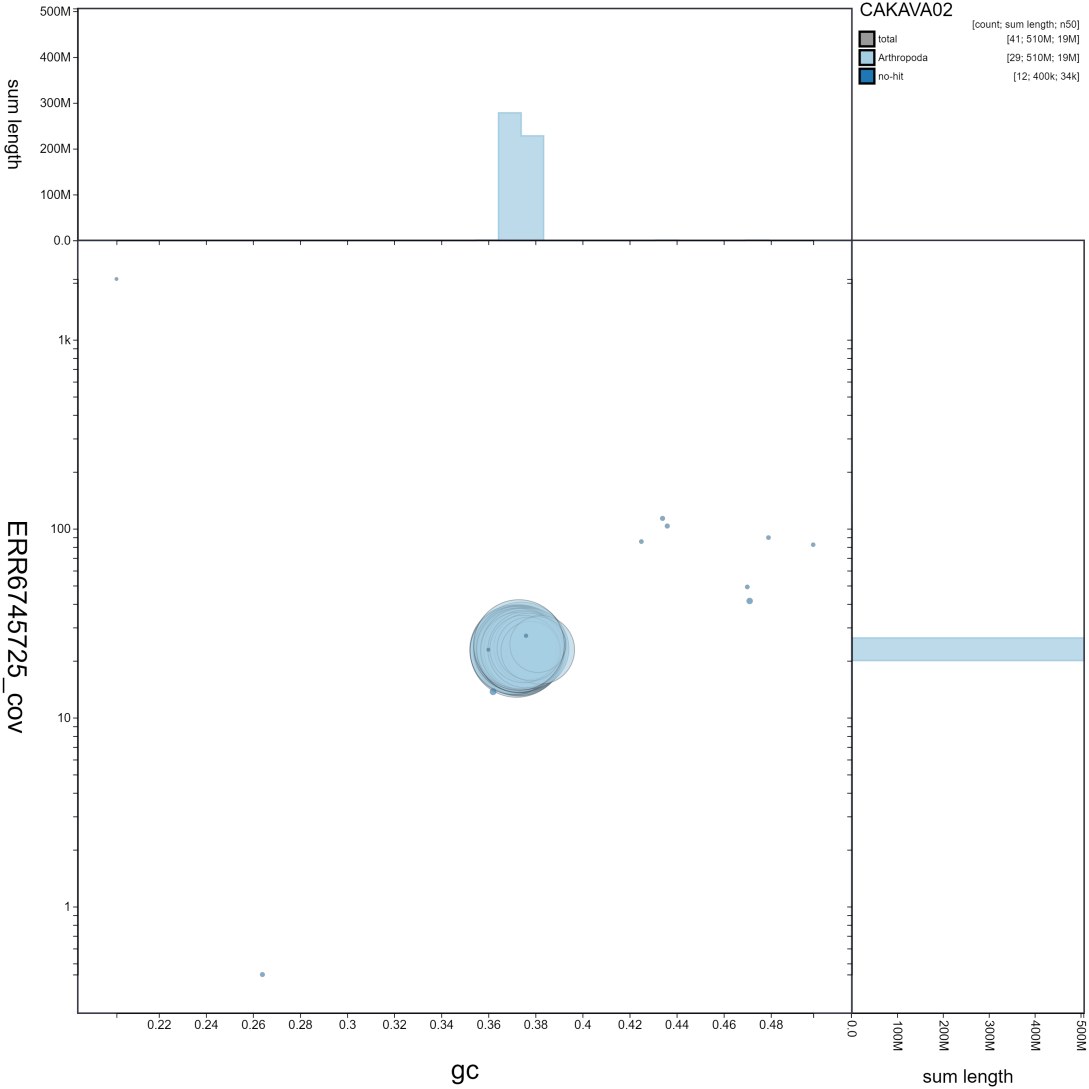
Genome assembly of *Erebia ligea*, ilEreLige1.2: GC coverage. BlobToolKit GC-coverage plot. Scaffolds are coloured by phylum. Circles are sized in proportion to scaffold length. Histograms show the distribution of scaffold length sum along each axis. An interactive version of this figure is available at https://blobtoolkit.genomehubs.org/view/ilEreLige1.2/dataset/CAKAVA02/blob.

**Figure 4.**
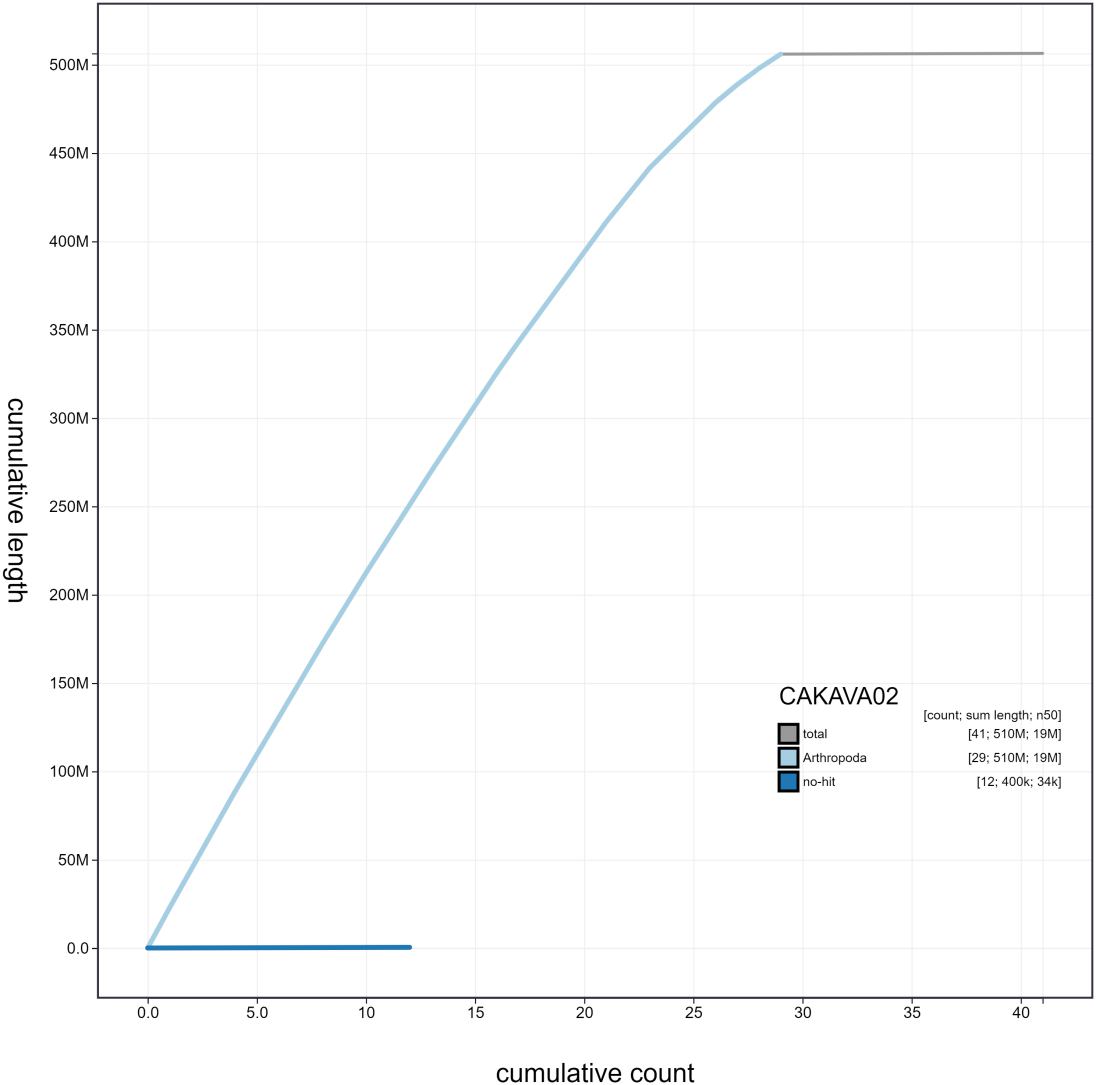
Genome assembly of *Erebia ligea*, ilEreLige1.2: cumulative sequence. BlobToolKit cumulative sequence plot. The grey line shows cumulative length for all scaffolds. Coloured lines show cumulative lengths of scaffolds assigned to each phylum using the buscogenes taxrule. An interactive version of this figure is available at https://blobtoolkit.genomehubs.org/view/ilEreLige1.2/dataset/CAKAVA02/cumulative.

**Figure 5.**
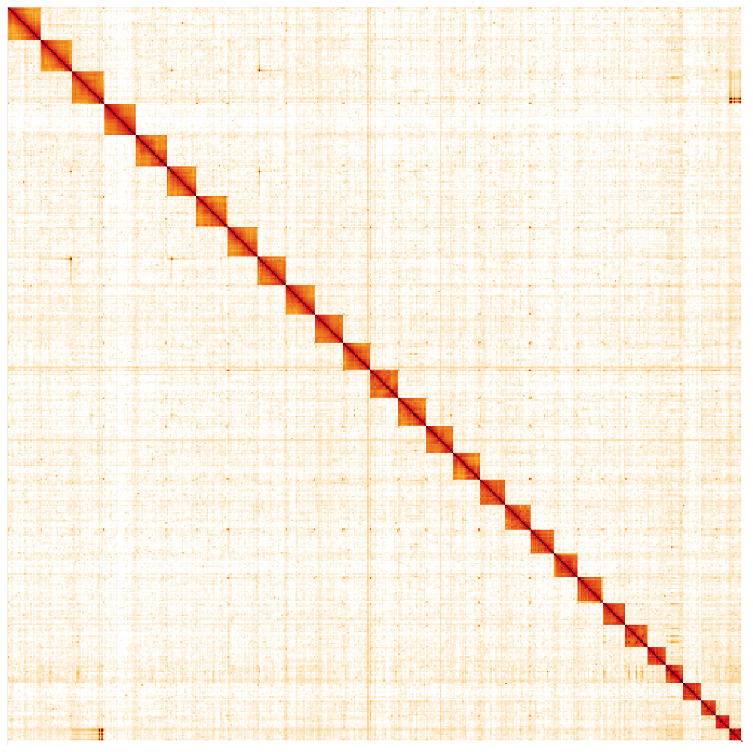
Genome assembly of *Erebia ligea*, ilEreLige1.2: Hi-C contact map. Hi-C contact map of the ilEreLige1.2 assembly, visualised in HiGlass. Chromosomes are shown in size order from left to right and top to bottom. The interactive Hi-C map can be viewed at https://genome-note-higlass.tol.sanger.ac.uk/l/?d=L3267sJjSyakmh-bayAgPg.

**Table 2.** Chromosomal pseudomolecules in the genome assembly of *Erebia ligea*, ilEreLige1.2.

INSDC accession	Chromosome	Size (Mb)	GC%
OU785219.1	1	22.72	37.2
OU785220.1	2	22.11	37.3
OU785221.1	3	22.01	37.4
OU785223.1	4	21.42	37.3
OU785224.1	5	20.97	37.3
OU785225.1	6	20.77	37.1
OU785226.1	7	20.52	37.1
OU785227.1	8	20.11	37.4
OU785228.1	9	20.06	37.3
OU785229.1	10	19.31	37.4
OU785230.1	11	19.22	37.3
OU785231.1	12	19.15	37.2
OU785232.1	13	18.89	37.3
OU785233.1	14	18.5	37.4
OU785234.1	15	18.36	37.3
OU785235.1	16	17.54	37.4
OU785236.1	17	17.22	37.7
OU785237.1	18	16.82	37.2
OU785238.1	19	16.82	37.4
OU785239.1	20	16.64	37.5
OU785240.1	21	15.51	37.3
OU785241.1	22	15.26	37.6
OU785242.1	23	12.47	37.7
OU785243.1	24	12.37	38.2
OU785244.1	25	11.94	37.5
OU785245.1	26	10.28	37.7
OU785246.1	27	9.22	37.8
OU785247.1	28	8.14	38.1
OU785222.1	Z	21.64	37.3
OU785248.1	MT	0.02	20.2
-	Unplaced	0.39	41.4

## Methods

### Sample acquisition and nucleic acid extraction

A single male *E. ligea* specimen (ilEreLige1, genome assembly, HiC) was collected from Borzont, Joseni, Harghita, Romania (latitude 46.664, longitude 25.317) using a handnet by Konrad Lohse, Dominik Laetsch (both University of Edinburgh) and Alex Hayward (University of Exeter). The sample was identified by Roger Vila (Institut de Biologia Evolutiva, Barcelona) and snap-frozen from live in a dry shipper.

DNA was extracted at the Scientific Operations Core, Wellcome Sanger Institute. The ilEreLige1 sample was weighed and dissected on dry ice with tissue set aside for Hi-C sequencing. Whole organism tissue was disrupted by manual grinding with a disposable pestle. Fragment size analysis of 0.01–0.5 ng of DNA was then performed using an Agilent FemtoPulse. High molecular weight (HMW) DNA was extracted using the Qiagen MagAttract HMW DNA extraction kit. Low molecular weight DNA was removed from a 200-ng aliquot of extracted DNA using 0.8X AMpure XP purification kit prior to 10X Chromium sequencing; a minimum of 50 ng DNA was submitted for 10X sequencing. HMW DNA was sheared into an average fragment size between 12–20 kb in a Megaruptor 3 system with speed setting 30. Sheared DNA was purified by solid-phase reversible immobilisation using AMPure PB beads with a 1.8X ratio of beads to sample to remove the shorter fragments and concentrate the DNA sample. The concentration of the sheared and purified DNA was assessed using a Nanodrop spectrophotometer and Qubit Fluorometer and Qubit dsDNA High Sensitivity Assay kit. Fragment size distribution was evaluated by running the sample on the FemtoPulse system.

### Sequencing

Pacific Biosciences HiFi circular consensus and 10X Genomics read cloud DNA sequencing libraries were constructed according to the manufacturers’ instructions. Sequencing was performed by the Scientific Operations core at the WSI on Pacific Biosciences SEQUEL II (HiFi) and Illumina HiSeq X (10X) instruments. Hi-C data were also generated from remaining whole organism tissue of ilEreLige1 using the Arima v1 Hi-C kit and sequenced on an Illumina HiSeq X (10X) instrument.

### Genome assembly

Assembly was carried out with Hifiasm ( Cheng *et al.*, 2021); haplotypic duplication was identified and removed with purge_dups ( Guan *et al.*, 2020). One round of polishing was performed by aligning 10X Genomics read data to the assembly with longranger align, calling variants with freebayes ( Garrison & Marth, 2012). The assembly was then scaffolded with Hi-C data ( Rao *et al.*, 2014) using SALSA2 ( Ghurye *et al.*, 2019). The assembly was checked for contamination and corrected using the gEVAL system ( Chow *et al.*, 2016) as described previously ( Howe *et al.*, 2021). Manual curation ( Howe *et al.*, 2021) was performed using gEVAL, HiGlass ( Kerpedjiev *et al.*, 2018) and Pretext. The mitochondrial genome was assembled using MitoHiFi ( Uliano-Silva *et al.*, 2021), which performed annotation using MitoFinder ( Allio *et al.*, 2020). The genome was analysed and BUSCO scores generated within the BlobToolKit environment ( Challis *et al.*, 2020). Table 3 contains a list of all software tool versions used, where appropriate.

**Table 3.** Software tools used.

Software tool	Version	Source
Hifiasm	0.12	Cheng *et al.,* 2021
purge_dups	1.2.3	Guan *et al.,* 2020
SALSA2	2.2	Ghurye *et al.,* 2019
longranger align	2.2.2	https://support.10xgenomics.com/ genome-exome/software/pipelines/ latest/advanced/other-pipelines
freebayes	1.3.1-17- gaa2ace8	Garrison & Marth, 2012
MitoHiFi	1	Uliano-Silva *et al.,* 2021
HiGlass	1.11.6	Kerpedjiev *et al.,* 2018
PretextView	0.1.x	https://github.com/wtsi-hpag/ PretextView
BlobToolKit	3.0.5	Challis *et al.,* 2020

## Data Availability

European Nucleotide Archive: *Erebia ligea* (Arran brown). Accession number PRJEB42125; https://identifiers.org/ena.embl/PRJEB42125. The genome sequence is released openly for reuse. The *E. ligea* genome sequencing initiative is part of the Darwin Tree of Life (DToL) project. All raw sequence data and the assembly have been deposited in INSDC databases. Raw data and assembly accession identifiers are reported in Table 1.

## Author information

Members of the Wellcome Sanger Institute Tree of Life programme are listed here: https://doi.org/10.5281/zenodo.6866293.

Members of Wellcome Sanger Institute Scientific Operations: DNA Pipelines collective are listed here: https://doi.org/10.5281/zenodo.5746904.

Members of the Tree of Life Core Informatics collective are listed here: https://doi.org/10.5281/zenodo.6125046.

Members of the Darwin Tree of Life Consortium are listed here: https://doi.org/10.5281/zenodo.6418363.

## References

[ref-1] Allio R Somaker-Bastos A Romiguier J : MitoFinder: Efficient Automated Large-Scale Extraction of Mitogenomic Data in Target Enrichment Phylogenomics. *Mol Ecol Resour.* 2020;20(4):892–905. 10.1111/1755-0998.13160 32243090 PMC7497042

[ref-2] Challis R Richards E Rajan J : BlobToolKit - interactive quality assessment of genome assemblies. *G3 (Bethesda).* 2020;10(4):1361–74. 10.1534/g3.119.400908 32071071 PMC7144090

[ref-3] Cheng H Concepcion GT Feng X : Haplotype-Resolved *de Novo* Assembly Using Phased Assembly Graphs with Hifiasm. *Nat Methods.* 2021;18(2):170–75. 10.1038/s41592-020-01056-5 33526886 PMC7961889

[ref-4] Chow W Brugger K Caccamo M : gEVAL — a Web-Based Browser for Evaluating Genome Assemblies. *Bioinformatics.* 2016;32(16):2508–10. 10.1093/bioinformatics/btw159 27153597 PMC4978925

[ref-5] de Vos JM Augustijnen H Bätscher L : Speciation through Chromosomal Fusion and Fission in Lepidoptera. *Philos Trans R Soc Lond B Biol Sci.* 2020;375(1806):20190539. 10.1098/rstb.2019.0539 32654638 PMC7423279

[ref-6] Dubatolov VV Korshunov YP Gorbunov PY : A Review of the Erebia ligea-Complex (Lepidoptera, Satyridae) from Eastern Asia. *Lepidoptera Science.* 1998;49(3):177–93. 10.18984/lepid.49.3_177

[ref-7] Federley H : Chromosomenzahlen Finnländischer Lepidopteren: I. Rhopalocera. *Hereditas.* 1938;24(4):397–464.

[ref-8] Fichefet V Barbier Y Baugnée JY : Papillons de Jour de Wallonie: (1985-2007).2008. Reference Source

[ref-9] Garrison E Marth G : Haplotype-Based Variant Detection from Short-Read Sequencing.2012; arXiv: 1207.3907. 10.48550/arXiv.1207.3907

[ref-10] Ghurye J Rhie A Walenz BP : Integrating Hi-C Links with Assembly Graphs for Chromosome-Scale Assembly. *PLoS Comput Biol.* 2019;15(8):e1007273. 10.1371/journal.pcbi.1007273 31433799 PMC6719893

[ref-11] Guan D McCarthy SA Wood J : Identifying and Removing Haplotypic Duplication in Primary Genome Assemblies. *Bioinformatics.* 2020;36(9):2896–98. 10.1093/bioinformatics/btaa025 31971576 PMC7203741

[ref-12] Howe K Chow W Collins J : Significantly Improving the Quality of Genome Assemblies through Curation. *GigaScience.* 2021;10(1):giaa153. 10.1093/gigascience/giaa153 33420778 PMC7794651

[ref-13] Kerpedjiev P Abdennur N Lekschas F : HiGlass: Web-Based Visual Exploration and Analysis of Genome Interaction Maps. *Genome Biol.* 2018;19(1):125. 10.1186/s13059-018-1486-1 30143029 PMC6109259

[ref-14] Kleckova I Konvicka M Klecka J : Thermoregulation and Microhabitat Use in Mountain Butterflies of the Genus *Erebia*: Importance of Fine-Scale Habitat Heterogeneity. *J Therm Biol.* 2014;41:50–58. 10.1016/j.jtherbio.2014.02.002 24679972

[ref-15] Kudrna O Pennerstorfer J Lux K : Distribution Atlas of European Butterflies and Skippers.Schwanfeld: Wiss. Verl. Peks.2015. Reference Source

[ref-16] Manni M Berkeley MR Seppey M : BUSCO Update: Novel and Streamlined Workflows along with Broader and Deeper Phylogenetic Coverage for Scoring of Eukaryotic, Prokaryotic, and Viral Genomes. *Mol Biol Evol.* 2021;38(10):4647–54. 10.1093/molbev/msab199 34320186 PMC8476166

[ref-17] Rao SS Huntley MH Durand NC : A 3D Map of the Human Genome at Kilobase Resolution Reveals Principles of Chromatin Looping. *Cell.* 2014;159(7):1665–80. 10.1016/j.cell.2014.11.021 25497547 PMC5635824

[ref-18] Robinson R : Lepidoptera Genetics.Oxford: Pergamon Press,1971. Reference Source

[ref-19] Saitoh K Abe A : The Chromosomes of Erebia ligea Rishirizana. *Nota Lepidopterol.* 1997;20(3/4):326–29. Reference Source

[ref-20] Salmon MA : Further Observations of the Erebia ligea (Linnaeus) and Other Controversies. *The Entomologist’s Record and Journal of Variation.* 1995;107:117.

[ref-21] Tolman T Lewington R : Collins Butterfly Guide: The Most Complete Guide to the Butterflies of Britain and Europe.HarperCollins Publishers Ltd.2008. Reference Source

[ref-22] Uliano-Silva M Nunes JGF Krasheninnikova K : marcelauliano/MitoHiFi: mitohifi_v2.0.2021. 10.5281/zenodo.5205678

[ref-23] van Swaay CAM Cuttelod A Collins S : European Red List of Butterflies.2010;47. 10.2779/83897

[ref-24] Warren BCS : On the Evolution of Subspecies, as Demonstrated by the Alternation of Variability Existing in the Subspecies of the Genus *Erebia* (Lepidoptera). *Zool J Linn Soc.* 1937;40(271):305–23. 10.1111/j.1096-3642.1937.tb01683e.x

[ref-25] Zakharova EY Tatarinov AG : Chrono-geographical Approach to Analysis of Variability of Bicyclic *Erebia ligea* (L.) (Lepidoptera: Satyridae) Species in the Urals. *Contemp Probl Ecol.* 2016;9(3):272–81. 10.1134/S1995425516030173

