# Supplementary material for: The genome sequence of the Arran brown, Erebia ligea (Linnaeus, 1758)
Source: Wellcome Open Res. 2022 Oct 13;7:259. [Version 1] doi: 10.12688/wellcomeopenres.18115.1 (PMC10280028; doi:10.12688/wellcomeopenres.18115.1)
Supplement: Peer review history

# Peer review: Reviewer response for version 1

**DOI:** 10.21956/wellcomeopenres.20086.r60428

**Published:** 2023-06-19

**Authors:** Manuela Lopez Villavicencio

**Affiliations:** 1 Museum National d'Histoire Naturelle Reseau des bibliotheques du museum, Paris, Île-de-France, France

This article reports the genome assembly of a male individual of the very widespread butterfly species *Erebia ligea*. The assembly included the autosomes, the Z sexual chromosome and the mitochondrial genome.

 The article is clearly written and pleasant to read. It shows convincing evidences for a high-quality assembly based on BUSCO scores. The methods for genome assembly, quality test and Hi-C scaffolding are relevant and up-to-date. There are sufficient details of methods and materials provided to allow replication by others.

 It is important to highlight that the assembly sequence was assigned to 40 chromosomal-level scaffolds, representing 28 autosomes plus the sexual Z chromosome and this is relevant because previous karyotypic analysis have found different chromosomal numbers for this species.  

 As I am interested in butterfly genomes, I would have loved to have other interesting information as genome-wide level of heterozygosity (estimated with jellyfish and genomescope).

 Overall, I think the release of this well-assembled and annotated genome is a very useful contribution and I recommend the indexing of this article.

Are sufficient details of methods and materials provided to allow replication by others?

Yes

Is the rationale for creating the dataset(s) clearly described?

Yes

Are the datasets clearly presented in a useable and accessible format?

Yes

Are the protocols appropriate and is the work technically sound?

Yes

Reviewer Expertise:

Evolutionary biology, Genomics

I confirm that I have read this submission and believe that I have an appropriate level of expertise to confirm that it is of an acceptable scientific standard.

# Peer review: Reviewer response for version 1

**DOI:** 10.21956/wellcomeopenres.20086.r53486

**Published:** 2022-12-14

**Authors:** Markus Moest

**Affiliations:** 1 Department of Ecology, University of Innsbruck, Innsbruck, Austria

Lohse et al. present a high-quality reference genome for a common Eurasian butterfly species. The quality of the assembly is impressive and represents a very valuable resource for future studies on biogeography and population genomics of the species as well as speciation research in the genus Erebia. 

 I only have few minor comments/questions:

**Background:**

 “relatively low altitudes”… I would suggest to provide an approximate altitudinal range or range of altitudinal zones that are inhabited by this species

 “in some northern localities it is recorded only every second year”...is the entire population really only present every other year or should this just mean that the completion of development can take up to 2 years?

**Genome Sequence Report:**

 "sequence was assigned to 40 chromosomal-level scaffolds, representing 28 autosomes (numbered by sequence length), and the Z sex chromosome"... this section and the fact that in the legend in Figure2 41 scaffolds are indicated confuses me. My interpretation of "chromosomal-level scaffolds" is 1 scaffold = 1 chromosome, but that does not the seem to be the case here (40 scaffolds = 28 autosomes + 1 Z) - could well be that my interpretation is the problem here. The 40 scaffolds in the text and the 41 scaffolds in Figure2 are maybe due to including/excluding the mitochondrial scaffold but I think this is not explicitly stated.

**Methods:**

 Some more details on the specific tissues that were used for extraction would be helpful - e.g. whether only thorax or muscle tissues were used, whether the abdomen and guts were excluded, etc. as this information is relevant in order to assess the risk of potential contamination. Moreover, the description of the DNA extraction procedure is a bit unclear. For example, was the DNA used for PacBio sequencing first size selected in the same way as for the 10X sequencing, how much DNA was used for PacBio and how was the tissue set aside for Hi-C processed?

 "A **single male** *E. ligea* specimen (ilEreLige1, genome assembly, HiC) was collected from Borzont, Joseni, Harghita, Romania (latitude 46.664, longitude 25.317) using a handnet by **Konrad Lohse**, **Dominik Laetsch **(both University of Edinburgh) and **Alex Hayward** (University of Exeter)."...obviously an extraordinarily strong specimen...

 General comment to Methods in data notes (not a comment to the authors!):

 I think it would be useful to provide a link to a general but more detailed description of the - I assume standardised - sequencing methods used in the DToL project, if that's available. This would provide interested readers with some more details and background and the method sections in the notes can remain short, concise and focussed on deviations from the general protocol.

Are sufficient details of methods and materials provided to allow replication by others?

Yes

Is the rationale for creating the dataset(s) clearly described?

Yes

Are the datasets clearly presented in a useable and accessible format?

Yes

Are the protocols appropriate and is the work technically sound?

Yes

Reviewer Expertise:

Evolutionary Ecology

I confirm that I have read this submission and believe that I have an appropriate level of expertise to confirm that it is of an acceptable scientific standard.
